# Serum NLR combined with CA125 and HE4 improves the diagnostic and prognostic efficiency in patients with ovarian cancer

**DOI:** 10.3389/fonc.2024.1494051

**Published:** 2024-12-20

**Authors:** Yun Tian, Xiabing Li, Hongjian Zhang, Yaping Wang, Hongyu Li, Qiaohong Qin

**Affiliations:** ^1^ Gynecologic Oncology, The Third Affiliated Hospital of Zhengzhou University, Zhengzhou, Henan, China; ^2^ Zhengzhou Key Laboratory of Gynecological Oncology, The Third Affiliated Hospital of Zhengzhou University, Zhengzhou, Henan, China

**Keywords:** ovarian cancer, diagnosis, NLR, CA125, HE4, prognosis

## Abstract

**Background:**

Ovarian cancer (OC) represents a common neoplasm within the female reproductive tract. The prognosis for patients diagnosed at advanced stages is unfavorable, primarily attributable to the absence of reliable screening markers for early detection. An elevated neutrophil-to-lymphocyte ratio (NLR) serves as an indicator of host inflammatory response and has been linked to poorer overall survival (OS) across various cancer types; however, its examination in OC remains limited. This study seeks to identify combination diagnostic and prognostic markers for OC, aiming to improve diagnostic and prognostic efficacy, especially in the early stages.

**Methods:**

We analyzed the targeted biomarkers in a cohort of 104 OC patients and 100 controls, which comprised 50 patients with benign ovarian tumors and 50 healthy women, using enzyme-linked immunosorbent assay (ELISA) and complete blood counting (CBC). After validating the biomarker panel, we compared the expression levels of the biomarkers in OC patients with various clinical features to assess their relevance. A biomarker panel was developed and validated with an independent cohort of 70 OC patients and 60 controls, including 30 with benign ovarian tumors and 30 healthy women. We evaluated the diagnostic accuracy using the area under the receiver-operating characteristic (ROC) curve and overall survival analysis was used for prognosis.

**Results:**

The results from ELISA and CBC analyses indicated that the NLR was significantly higher in patients with OC. This elevation was especially notable in those with advanced stages of the disease, lymph node metastasis, and ascites. The diagnostic performance of the NLR, when combined with CA125 and HE4, outperformed each marker used individually, especially when compared to the traditional combination of CA125 and HE4. Importantly, we observed similar results in patients with early-stage ovarian cancer and those with low levels of CA125 and HE4. In addition, these results suggest that NLR combined with CA125 and HE4 levels in OC patients have significant prognostic value.

**Conclusions:**

The effective combination of serum NLR, CA125, and HE4 significantly enhances diagnostic efficiency in patients with OC. Serum NLR, CA125, and HE4 levels were identified as independent prognostic markers for OC.

## Background

Ovarian cancer (OC) is one of the common malignant tumors of female reproductive system, the incidence is second only to cervical cancer and endometrium cancer, but has the highest mortality rate (1). The prognosis for advanced OC is often poor due to extensive abdominal implant metastases, lymph node metastases and systemic metastases (2, 3). However, due to the insidious nature of the early lesions, there is no significant symptoms in early stage, so far there is no effective early stage screening indicator (4). Most patients are already in the middle or advanced stages (Figo stage III-IV) at the time of diagnosis (5). Despite advances in surgery, chemotherapy and other adjuvant treatments, the five-year survival rate for advanced OC is still as low as 40% (3). The search for effective early diagnosis and markers indicating the extent of OC progression is important for the treatment and prognosis of patients with OC, so as to timely diagnosis and treatment, prolong the survival rate of patients, and improve the quality of patients’ life.

Several tumor biomarkers have been evaluated in OC, in the early 1980’s, the Carbohydrate Antigen 125 (CA125) was first described (6). Due to increased CA125 levels are also reported in other physiological or pathological conditions, such as menstruation, pregnancy, endometriosis and inflammatory diseases of the peritoneum (7), in cases of OC, serum CA125 level may be elevated, but this marker has a low sensitivity in the early stages of OC (8). Therefore, there have been attempts to find other biomarkers that can complement or replace CA125, human epididymal protein 4 (HE4) was found to be a reliable biological marker for detecting OC (level of evidence (9), and it has better specificity than CA125 (10). Although the specificity of these markers is rather reliable, they are not very sensitive, so the combination of these markers and the menopausal status of patients led to the proposition of the risk of ovarian malignancy algorithm (ROMA) to predict OC. However, there have been discrepancies regarding the reported diagnostic performances of CA125, HE4, and ROMA in previous studies (10).

In addition, the detection of various genetic mutations has been reported in ovarian cancer, and inhibitors of several related pathways have been developed, whose anti-tumor potential is currently being evaluated in different clinical trials. BRCA1/2 mutations are at the heart of homologous recombination repair deficiency (HRD) in ovarian cancer, and mismatch repair (MMR) detection in ovarian cancer is commonly associated with non-serous ovarian cancer and is often associated with Lynch syndrome. A large proportion of ovarian cancer patients have HRD, and because HRD is associated with response to platinum chemotherapy and PARP inhibitors, it can influence treatment decisions for first - and second-line therapies (11). Mutations in genes encoding the RAS-RAF-MEK-ERK pathway protein can be detected in ovarian cancer, and BRAF and MEK inhibitor combinations are more effective and safer for ovarian cancer, especially for low-grade serosal ovarian cancer (12). The search for new and effective diagnostic and prognostic biomarkers for ovarian cancer is critical.

Systemic inflammatory response plays a crucial role in all stages of tumor. Systemic inflammation can up-regulate cytokines and inflammatory mediators, inhibit apoptosis, initiate angiogenesis, reshape extracellular matrix and trigger DNA damage (13–15). Biological indicators of the severity of systemic inflammation include C-reactive protein, NLR, platelet to lymphocyte ratio(PLR), lymphocyte tomonocyte ratio(LMR), and lymphocyte tomonocyte ratio and platelet count. In recent years, inflammatory blood markers have become prognostic factors for different types of tumors, especially NLR (16). In NLR, the ratio of monocytes and neutrophils as immunosuppressive cells and lymphocytes as immunoactivator cells can reflect the systemic inflammatory state of patients. John-Olabode et al (17) found that NLR could be used as an independent prognostic factor for epithelial ovarian cancer. Nøst et al. (18) applied four indicators related to inflammation and immunity, namely systemic immune-inflammation index (SII), NLR, PLR, and LMR, to evaluate the risk of 17 cancers. SII, NLR and PLR were found to be positively correlated with the risk of 7 types of cancer. In studies of solid tumors, high NLR has been shown to increase in patients with advanced tumors with lymph node metastasis and is associated with poor prognosis (19, 20). In addition, different researchers have found that high NLR is a predictor of poor efficacy and recurrence of malignant tumors (10, 21–24). what’s more, it has been reported that NLR is elevated in epithelial ovarian cancer (25). In this study, we evaluated the expression difference of conventional ovarian cancer markers, NLR and combination indicators in ovarian cancer patients and healthy population and the diagnostic and prognostic efficacy in ovarian cancer, aiming to find new combination diagnostic and prognostic markers of ovarian cancer and improve the diagnostic and prognostic efficacy, especially in early stage.

## Materials and methods

### Study design

**Figure 1:**
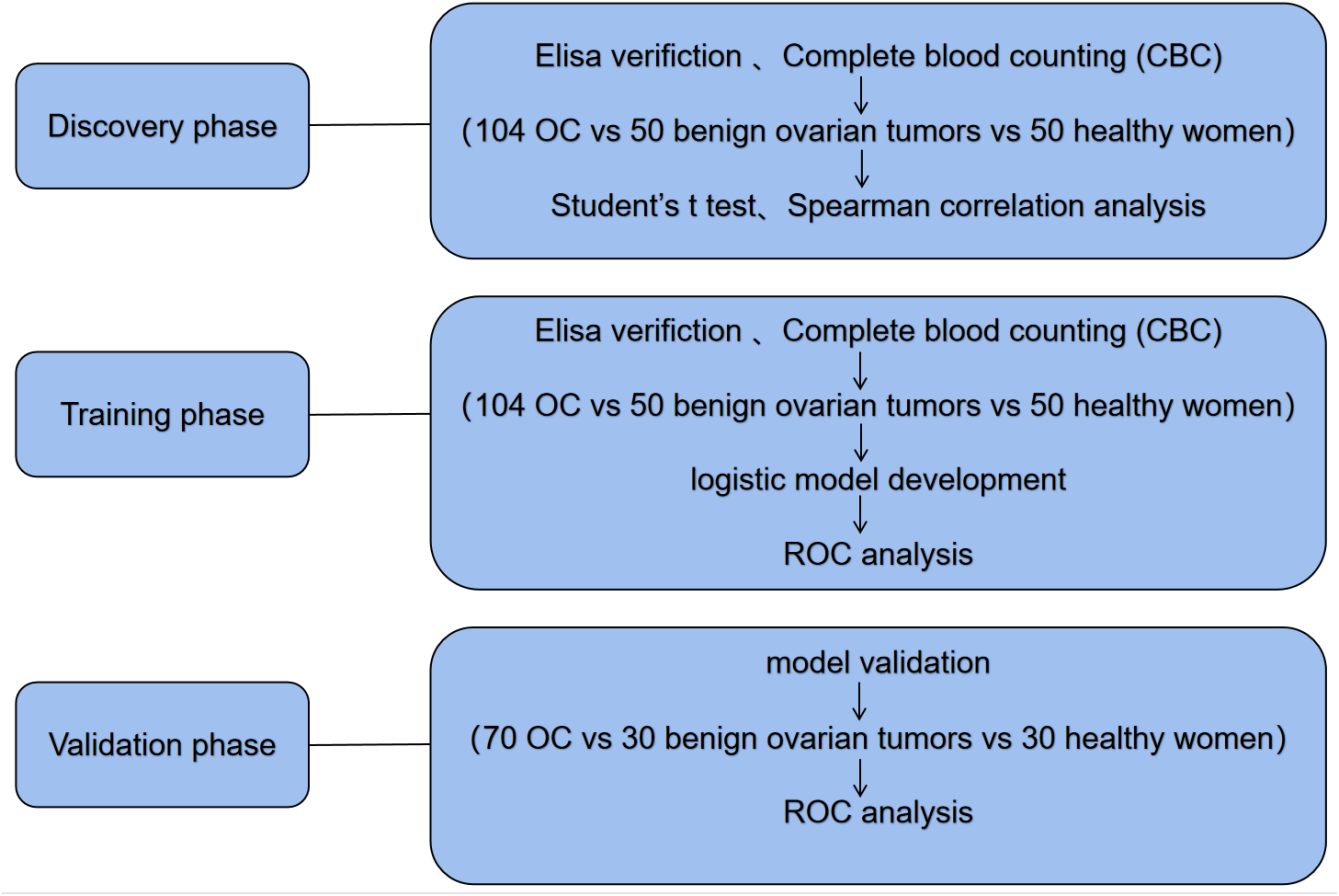
Design of the study (OC, Ovarian cancer).

This is a retrospective study. The entire research was comprised of three distinct phases: the discovery phase, the training phase, and the validation phase (**Figure 1**). During the discovery phase, we collected the targeted biomarkers by using a cohort consisting of 104 OC patients and 100 control group. In the control group, 50 patients with benign ovarian tumors and 50 healthy women were included. In the training group, we used a previously collected cohort of 104 OC patients with ovarian cancer and 100 controls to investigate whether it could be used as a tumor marker for early diagnosis of ovarian cancer. Then a logistic regression model was employed to establish the panel of targeted biomarkers. In order to enhance the assessment of the model’s diagnostic precision, we employed receiver-operating characteristic (ROC) curves. In the subsequent validation phase, the efficacy of the logistic model was assessed by distinguishing OC patients from a control group within a separate independent validation cohort comprising 70 OC patients and 60 controls, which included 30 individuals with benign ovarian tumors and 30 healthy women.

### Inclusion criteria

a. The operation was performed in our hospital with clear pathological diagnosis and complete clinicopathological data; b. Blood routine and CA125 and HE4 test results were obtained 1 week before surgery; c. Overall staging surgery was performed for patients with early ovarian malignancies, and tumor cell reduction was performed for patients with advanced ovarian malignancies.

### Exclusion criteria

a. The clinicopathological data were incomplete; b. Patients with other malignant tumors; c. Blood complications such as agranulocytosis and thrombocytopenia; d. The patients were treated with antibiotics, glucocorticoids, radiotherapy and chemotherapy before operation; e. Patients with an ongoing course of infection were excluded; f. Patients with autoimmune diseases; g. patients who had received treatments that could potentially elevate the NLR, such as granulocyte colony-stimulating factor, within one month prior to surgery.

### Patient selection

A total number of 334 patients was identified from the Third Affiliated Hospital of Zhengzhou University, spanning the period from June 2016 to June 2020. These patients were divided into three groups: the discovery phase, the training phase, and the validation phase. This cohort comprised 104 individuals classified within the malignant group, all of whom had undergone surgical procedures leading to a postoperative pathological diagnosis of ovarian cancer. The diagnosis of ovarian and ovarian benign cysts and normal ovarian tissue was made by at least two pathologists above the attending physician. The Ovarian cancer stage was classified based on the International Federation of Gynecology and Obstetrics (FIGO). These patients were receiving their surgical treatment and had not previously undergone radiotherapy or endocrine therapy. The NLR was established as the quotient of absolute neutrophil counts to absolute lymphocyte counts. A total of 50 patients diagnosed with benign ovarian conditions were chosen to constitute the benign group, while an additional 50 healthy women were selected for the control group. Comprehensive data regarding serum tumor markers and standard blood tests were available for all participants. We collected relevant clinical information and other overall survival data via medical records and telephone inquiries. The clinical characteristics of the individuals in each group are presented in the table below (**Table 1**). This study was approved by our medical ethics committee (ethics approval number:2023-174-01). In addition, we obtained written informed consent from each patient and provided accessible follow-up information.

**Table 1:** Clinicopathological data for OC patients and controls.

Characteristics	Discovery phase	Training phase	Validation phase
Normal			
Number	50	50	30
Age (mean ± s. d), years	51.4±11.0	51.4±11.0	51.3±11.2
Benign			
Number	50	50	30
Age (mean ± s. d), years	47.2±6.4	47.2±6.4	46.3±5.5
Pathology, n (%)			
Mature cystic teratoma	20(40.0)	20(40.0)	12(40.0)
Chocolate cyst	30(60.0)	30(60.0)	18(60.0)
Tumor			
Number	104	104	70
Age, (mean ± s. d), years	50.9±11.7	50.9±11.7	50.8±12.1
Figo stage, n (%)			
I-	35(33.7)	35(33.7)	24(34.3)
II	13(12.5)	13(12.5)	8(11.4)
III	52(50)	52(50)	36(51.4)
IV	4 (3.8)	4 (3.8)	2(2.9)
N, n (%)			
Yes	31(29.8)	31(29.8)	21(30)
No	73(70.2)	73(70.2)	49(70)
Ascites, n (%)			
Yes	73(70.2)	73(70.2)	48(68.6)
No	31(29.8)	31(29.8)	22(31.4)
Menopause, n (%)			
Yes	52(50)	52(50)	35(50)
No	52(50)	52(50)	35(50)
Pathology, n (%)			
Serous cystadenoma	67(64.4)	67(64.4)	44(62.9)
Mucinous cystadenoma	18(17.3)	18(17.3)	13(18.5)
Clear cell adenocarcinoma	13(12.5)	13(12.5)	9(12.9)
Endometrioid carcinoma	6 (5.8)	6 (5.8)	4(5.7)
Tumor size, n (%)			
<9cm	48(46.2)	48(46.2)	33(47.1)
≥9cm	56(53.8)	56(53.8)	37(52.9)
CA125, n (%)			
Normal	16(15.4)	16(15.4)	12(17.1)
High	88(84.6)	88(84.6)	58(82.9)
HE4, n (%)			
Normal	47(45.2)	47(45.2)	32(45.7)
High	57(54.8)	57(54.8)	38(54.3)
CEA, n (%)			
Normal	95(91.3)	95(91.3)	62(88.6)
High	9 (8.7)	9 (8.7)	8(11.4)
AFP, n (%)			
Normal	100(96.2)	100(96.2)	67(95.7)
High	4(3.8)	4(3.8)	3(4.3)
NLR (mean ± s. d)			
Normal	62(59.6)	62(59.6)	41(58.6)
High	42(40.3)	42(40.3)	29(41.4)

N, lymph node metastases.

### ELISA

Fasting venous blood samples were obtained in volumes of 3-4ml, subsequently subjected to centrifugation at a speed of 3000 revolutions per minute for a duration of 10 minutes, after which the serum was extracted for analysis. The levels of serum CA125, HE4, alpha-fetoprotein (AFP), and carcinoembryonic antigen (CEA) were measured using the ELISA technique, employing the Fully Automatic Chemiluminescence Immunoassay Analyzer (cobas 8000 e 801) along with its corresponding test kits. The procedures were meticulously conducted in accordance with the specified operational guidelines.

### CBC

CBC with automated differential counts was performed for all patients and the healthy controls using Fully automatic blood cell analyzer (BC-7500[NR] CRP). On the day of admission or the morning of the second day, 2 ml of peripheral fasting blood was collected from the cubital vein, and the serum was separated for analysis. CBC test was performed within 4 hours. The NLR was calculated by dividing the absolute neutrophil count (ANC) by the absolute lymphocyte count (ALC).

### Statistical analyses

Statistical analyses were conducted using SPSS version 26.0 and GraphPad Prism8.0.2 software. Comparisons among various groups were performed employing the Student’s t-test, Chi-square test, and one-way ANOVA. Additionally, Spearman correlation analysis, as well as both univariate and multivariate logistic regression models, were executed. A diagnostic model was established through logistic regression analysis. ROC curves were generated by plotting sensitivity against specificity, and the areas under the curves (AUC) were evaluated using the Hanley and McNeil method. A p-value of less than 0.05 was deemed statistically significant (*p<0.05, **p<0.01, ***p<0.001, ****p<0.0001).

## Results

### Clinical features and laboratory data

The clinical data of patients in this study are shown in **Table 1**. In the discovery phases and training phases, the mean age of patients with normal ovaries was 51.4 years (SD = 11.0), those with benign ovarian cysts were 47.2 years (SD = 6.4), and those with ovarian cancer were 50.9 years (SD = 11.7). There were 52 cases of menopausal patients and 52 cases of premenopausal patients. There were 35 cases of FIGO stage I and 13 cases of FIGO stage II. There were 52 cases of FIGO stage III and 4 cases of FIGO stage IV. There were 67 cases of serous ovarian cancer, 18 cases of mucinous ovarian cancer, 13 cases of clear cell carcinoma and 6 cases of endometrioid adenocarcinoma. There were 31 cases of patients with lymph node metastasis and 73 cases of patients without lymph node metastasis. In the validation phases, the mean age of patients with normal ovaries was 51.3 years (SD = 11.2), those with benign ovarian cysts were 46.3 years (SD = 5.5), and those with ovarian cancer were 50.8 years (SD = 12.1). There were 35 menopausal patients and 35 premenopausal patients. There were 24 cases of FIGO stage I patients and 8 cases of FIGO stage II patients. There were 36 cases of FIGO stage III patients and 2 cases of FIGO stage IV patients. There were 44 cases of serous ovarian cancer, 13 cases of mucinous ovarian cancer, 9 cases of clear cell carcinoma and 4 cases of endometrioid adenocarcinoma. There were 21 cases of patients with lymph node metastasis and 49 cases of patients without lymph node metastasis.

### CA125, HE4 and NLR were high expression in discovery phases

**Figure 2:**
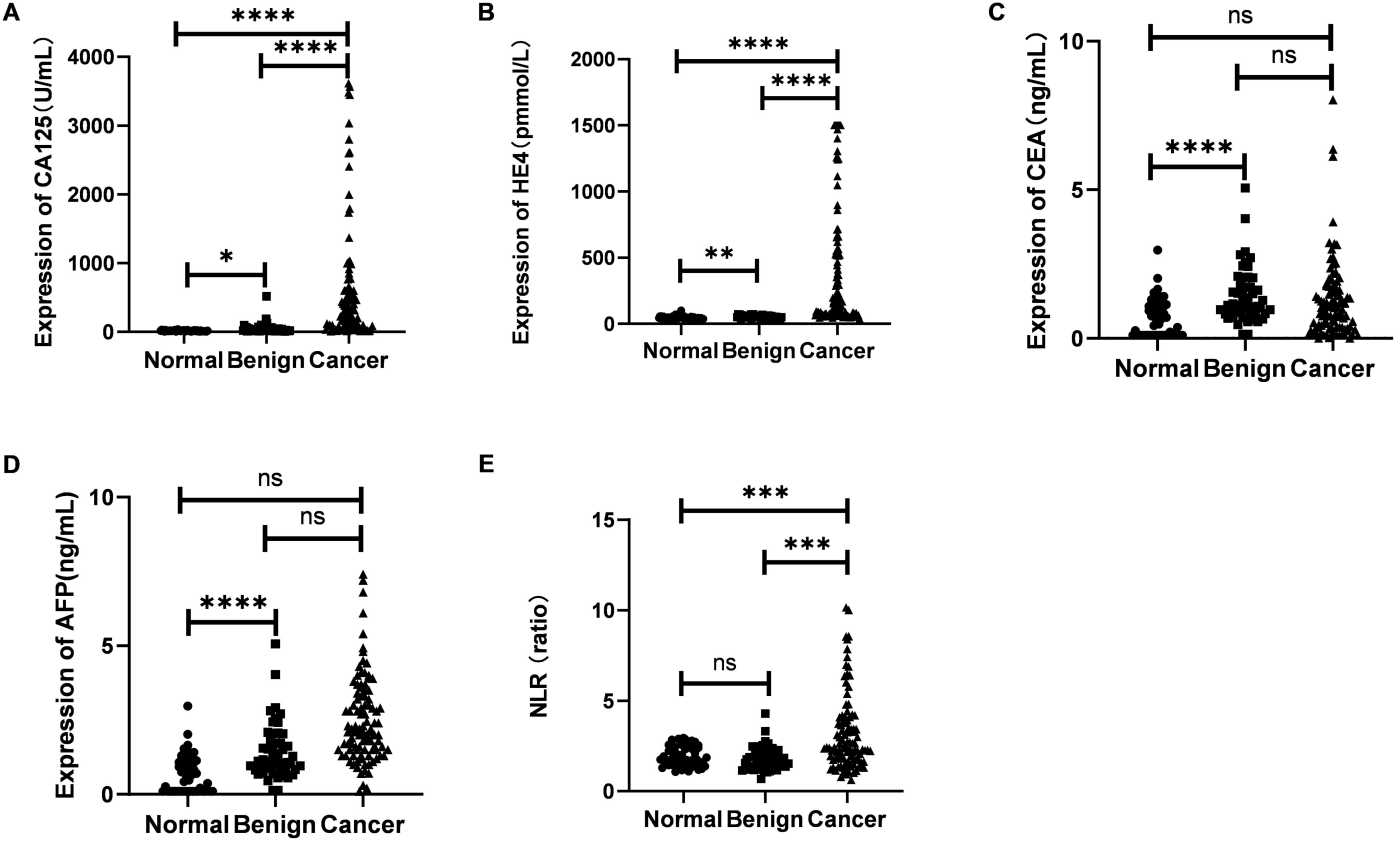
Evaluation of the expression of CA125 **(A)**, HE4 **(B)**, CEA **(C)**, AFP **(D)** and NLR **(E)** in sera from 104 OC patients and 50 benign ovarian tumors and 50 healthy women. Student’s t test was used. (ns ≥ 0.05; *p < 0.05; **p < 0.01; ***p < 0.001; ****p < 0.0001).

**Figure 3:**
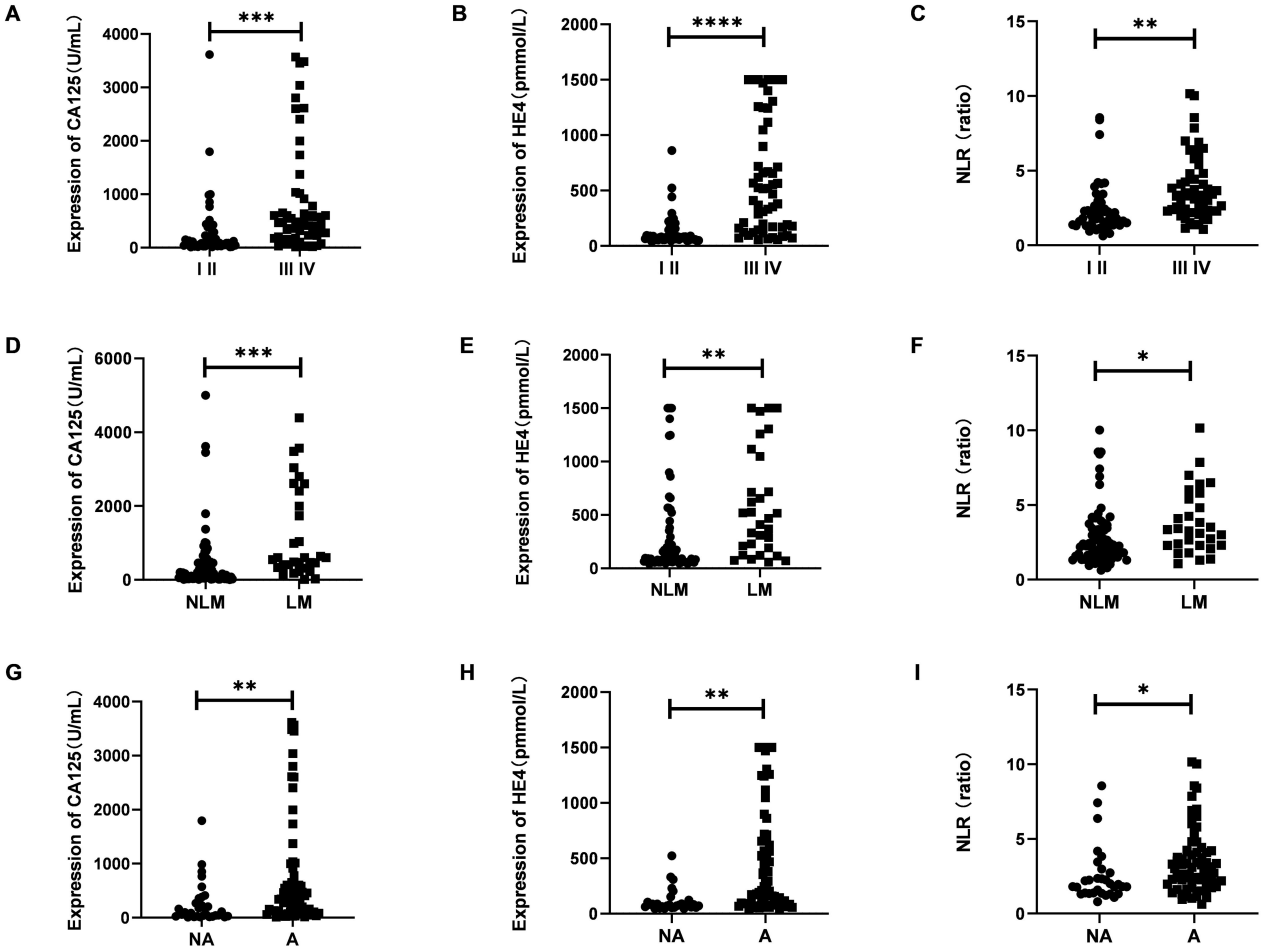
The expression of serumCA125, HE4 and NLR in different stage **(A–C)**, lymph node metastasis or not **(D–F)** and ascites or not **(G–I)**. Student’s t test was used. (I II, Figo I-II stages; III IV, Figo III-IV stages; NLM, without lymph node metastases; LM, with lymph node metastases; NA, without ascites; A, with ascites; *p < 0.05; **p < 0.01; ***p < 0.001; ****p < 0.0001).

**Figure 4:**
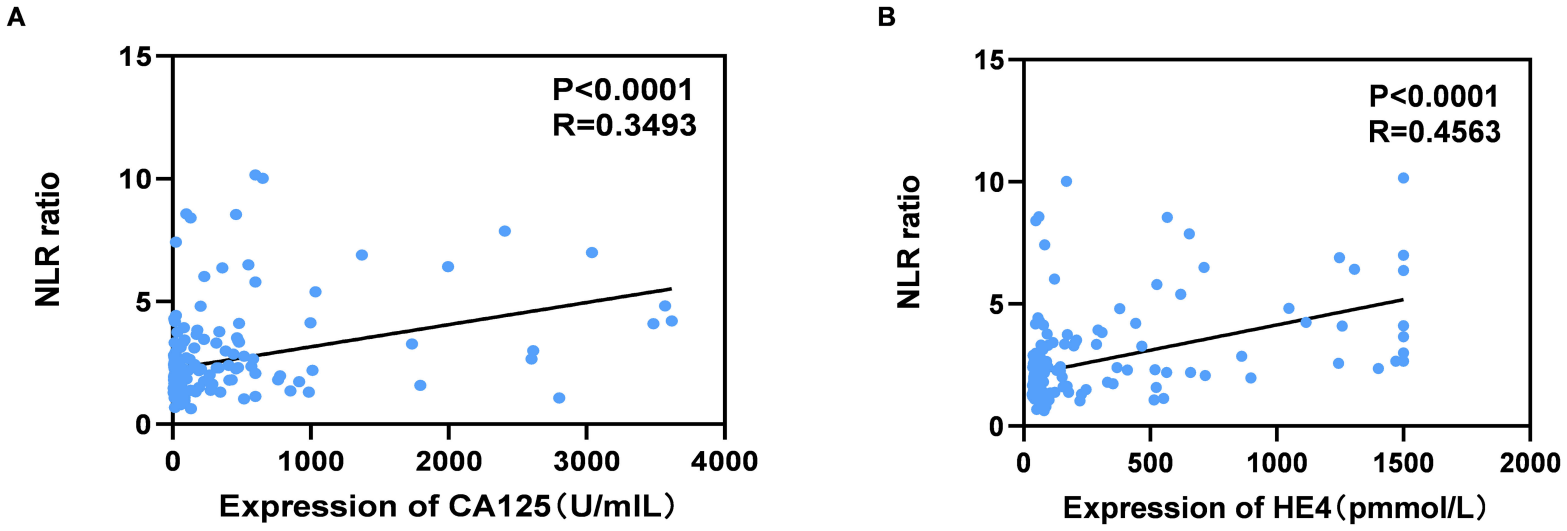
Correlation between serum levels of NLR and CA125 **(A)**, NLR and HE4 **(B)** in OC patients. Spearman correlation coefficient was calculated.

As illustrated in **Figure 2**, the serum levels of CA125 and HE4 in patients diagnosed with OC were markedly elevated compared to those observed in the control group (p<0.0001; **Figures 2A, B**). In contrast, no significant differences were detected in the serum concentrations of CEA and AFP between OC patients and controls (p≥0.05; **Figures 2C, D**). Additionally, the NLR was significantly higher in OC patients than in the control group (p<0.001; **Figure 2E**). Furthermore, levels of CA125, HE4, and NLR were found to be increased in OC patients presenting with advanced disease stages (p<0.001, p<0.0001, p<0.01; **Figures 3A–C**). Notably, CA125, HE4, and NLR levels were significantly elevated in patients with lymph node metastases compared to those without such metastases (p<0.001, p<0.01, p<0.05; **Figures 3D–F**), as well as in patients with ascites relative to those without ascites (p<0.01, p<0.01, p<0.05; **Figures 3G–I**). Spearman correlation analysis revealed a significant positive correlation between NLR and both CA125 and HE4 levels, respectively (r=0.3493, p=0.0001; r=0.4563, p=0.0001; **Figures 4A, B**).

### Relationship between serum NLR, CA125 and HE4 levels and clinicopathological parameters

The data presented in **Tables 2** and **3** indicate that elevated levels of NLR, CA125, and HE4, whether considered individually or in combination, were markedly correlated with factors such as advanced age, increased Figo T stage, elevated N stage, the occurrence of ascites, postmenopausal status, and greater tumor dimensions. Our findings indicate possible contributions of NLR, CA125, and HE4 in the advancement of OC.

**Table 2:** Comparison of high and low expression of serum CA125, HE4 and NLR in clinical parameters.

	Total	CA125(U/mL)		X2	p Value	HE4(pmmol/L)		X2	p Value	NLR(ratio)		X2	p Value
		High(>35)	Low(≤35)			High(>140)	Low(≤140)			High(≥3)	Low(<3)		
Age				0.065	0.798			13.117	<0.001***	12	30	4.084	0.043*
<50	42	36	6			14	28			30	32		
≥50	62	52	10			43	19						
Figo stage				2.033	0.154			27.663	<0.001***				
I-II	48	38	10			13	35			10	38	14.153	<0.001***
III-IV	56	50	6			44	12			32	24		
N				2.707	0.178			9.117	0.003**				
Yes	31	29	2			24	7			18	13	5.734	0.017*
No	73	59	14			33	40			24	49		
Ascites				6.319	0.027*			14.997	<0.001***				
Yes	73	66	7			49	24			36	37	8.113	0.004**
No	31	22	9			8	23			6	25		
Menopause				0.295	0.587			4.697	0.03*				
Yes	52	43	9			34	18			23	29	0.639	0.424
No	52	45	7			23	29			19	33		
Tumor size				3.885	0.049*			6.215	0.013*				
<9cm	48	37	11			20	28			18	30	0.308	0.579
≥9cm	56	51	5			37	19			24	32		

N, lymph node metastases; *p < 0.05; **p < 0.01; ***p < 0.001.

**Table 3:** Comparison of high and low expression of CA125 combined with HE4 and CA125, HE4 combined with NLR in clinical parameters.

	Total	CA125 combined with HE4		X2	p Value	CA125, HE4 combined with NLR		X2	p Value
		High	Low			High	Low		
Age				12.940	<0.001***			12.940	<0.001***
<50	42	30	12			30	12		
≥50	62	22	40			22	40		
Figo stage				26.155	<0.001***			30.333	<0.001***
I-II	48	37	11			38	10		
III-IV	56	15	41			14	42		
N				13.281	<0.001***			13.281	<0.001***
Yes	31	7	24			7	24		
No	73	45	28			45	28		
Ascites				13.281	<0.001***			13.281	<0.001***
Yes	73	28	45			28	45		
No	31	24	7			24	7		
Menopause				3.846	0.050			5.538	0.019*
Yes	52	21	31			20	32		
No	52	31	21			32	20		
Tumor size				7.583	0.006**			5.571	0.018*
<9cm	48	31	17			30	18		
≥9cm	56	21	35		22		34		

N, lymph node metastases; *p < 0.05; **p < 0.01; ***p < 0.001.

### CA125 and HE4 combined with NLR can improve the diagnostic efficiency in training phases

**Figure 5:**
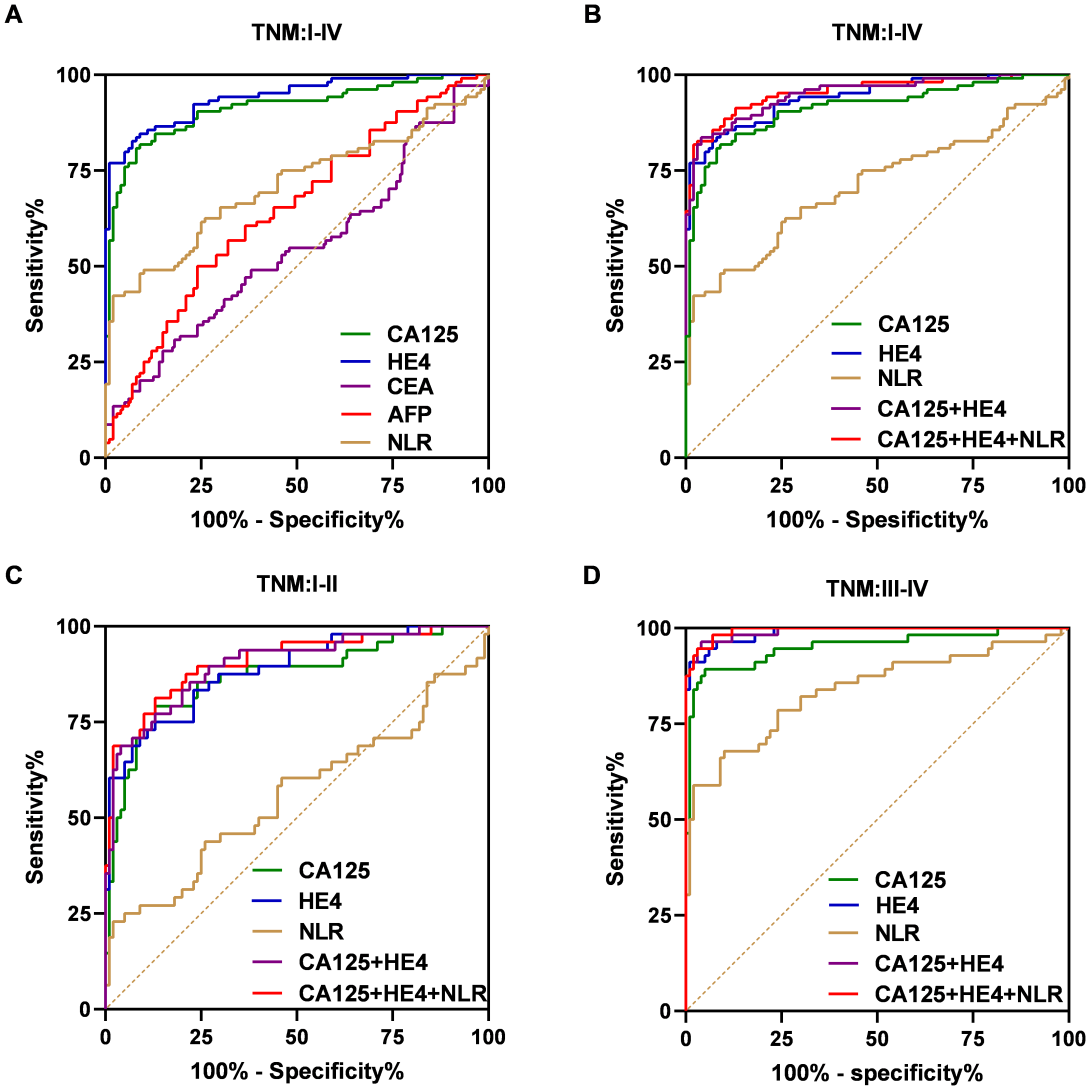
ROC curves for the markers to differentiate OC patients from the control group in the training dataset. ROC curves of the CA125, HE4, CEA, AFP and NLR for OC cases with overall stages (I–IV) **(A)**. ROC curves of the CA125, HE4, NLR, combination of the CA125 and HE4 and combination of the CA125, HE4 and NLR for OC cases with overall stages (I–IV) **(B)**, early stages (I–II) **(C)**, advanced stages (III–IV) **(D)**.

The effectiveness of serum NLR, CA125, and HE4 in differentiating OC patients from control subjects was assessed through the utilization of the ROC curve analysis. As shown in **Figure 5A**, the efficacy of CA125 (AUC=0.9129; cut-off value: 39.570; sensitivity: 80.77%; specificity: 92%) was superior to that of CEA (AUC=0.5423; cut-off value: 1.740; sensitivity: 27.88%; specificity: 85%). Similarly, HE4 (AUC=0.9408; cut-off value: 63.250; sensitivity: 76.92%; specificity: 99%) and NLR (AUC=0.7098; cut-off value: 2.031; sensitivity: 42.31%; specificity: 98%) also demonstrated better efficacy compared to AFP (AUC=0.6450; cut-off value: 2.295; sensitivity: 50%; specificity: 76%). The combination of CA125 and HE4 in a logistic regression model yielded a better screening efficacy (AUC=0.9494; cut-off value: 0.499; sensitivity: 83.65%; specificity: 96%) than using either marker alone (**Figure 5B**). When CA125, HE4 and NLR were combined in a logistic regression model, the screening efficacy improved further (AUC=0.9544; cut-off value: 0.376; sensitivity: 81.73%; specificity: 98%) compared to the combination of CA125 and HE4 alone (**Figure 5B**). The predicted probability of ovarian cancer diagnosis was calculated using the stepwise logistic regression model as follows:

logit (P) = −8.822 + 3.743 CA125 + 1.030 HE4.

logit (P) = 7.664 - 0.006 CA125 - 0.093 HE4 - 0.493 NLR.

Additionally, we evaluated how effectively the established biomarker panel differentiates early-stage OC patients from control subjects. Similar results were found in individuals with early-stage disease (I/II) (**Figure 5C**). As shown in **Figure 4C**, the AUC values for the NLR, CA125 and HE4 were 0.5598 (cut-off: 2.229; sensitivity: 22.92%; specificity: 98%), 0.8679 (cut-off: 39.570; sensitivity: 79.17%; specificity: 87%), and 0.8851 (cut-off: 59.240; sensitivity: 75%; specificity: 87%), respectively. Importantly, combining CA125 and HE4 produced an AUC of 0.9006 (cut-off: 0.499; sensitivity: 68.75%; specificity: 96%), exceeding the individual AUCs of these markers. Furthermore, integrating CA125, HE4 and NLR increased the AUC to 0.9083 (cut-off: 0.720; sensitivity: 81.25%; specificity: 87%). In advanced-stage patients, similar results were observed (**Figure 5D**). Moreover, the combination of CA125, HE4 and NLR demonstrated superior screening performance (AUC = 0.9939; cut-off: 0.402; sensitivity: 94.64%; specificity: 97%) compared to that observed in the early-stage cohort.

**Figure 6:**
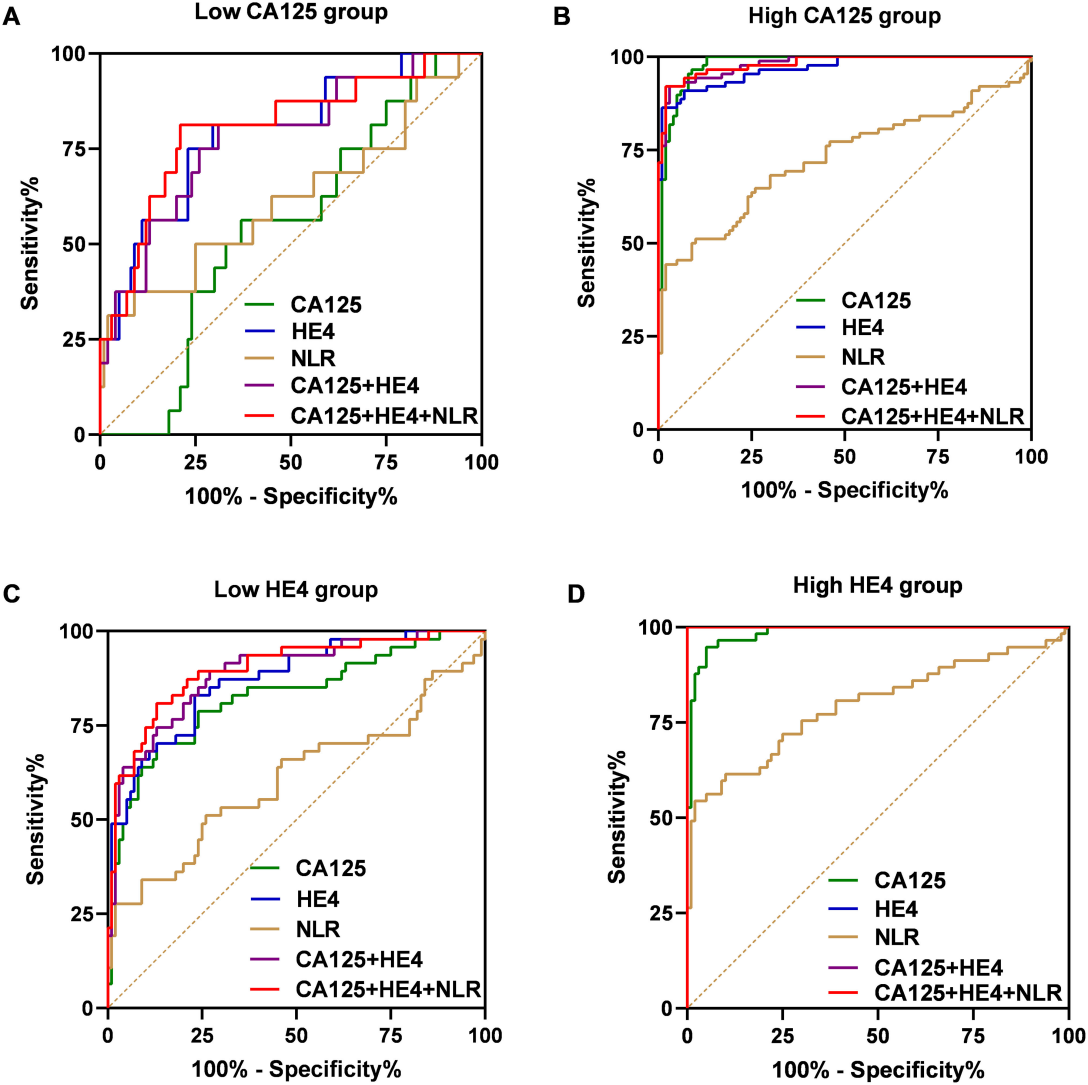
ROC curves of the CA125, HE4, NLR, combination of the CA125 and HE4 and combination of the CA125, HE4 and NLR for OC cases with low-CA125 level group **(A)**, high-CA125 level group **(B)**, low-HE4 level group **(C)**, high-CA125 level group **(D)**.

Subsequently, we assessed the diagnostic performance of the established panel based on the levels of CA125 and HE4 (**Figure 6**). In the group with low CA125 levels (≤35 U/mL), the AUC for the combination of CA125, HE4 and NLR was determined to be 0.8063, demonstrating a sensitivity of 68.75% and a specificity of 80% (**Figure 6A**). Conversely, in the High CA125 level group (>35 U/mL), the AUC for the same combination reached 0.9814, with a sensitivity of 92.05% and a specificity of 98% (**Figure 6B**). For the Low HE4 level group (≤140 pmol/L), the AUC for the combination of CA125, HE4 and NLR was found to be 0.8991, exhibiting a sensitivity of 80.85% and a specificity of 87% (**Figure 6C**). Notably, within these groups, the AUC for the combination of CA125, HE4 and NLR was the highest among the five evaluated markers. In the High HE4 level group (>140 pmol/L), the AUC for the combination of CA125, HE4 and NLR was recorded as 1, indicating both a sensitivity and specificity of 100% (**Figure 6D**). Additionally, the AUC for HE4 alone and the combination of CA125 and HE4 also yielded a value of 1 (HE4: sensitivity 100%; specificity 88%; CA125 + HE4: sensitivity 100%; specificity 88%; **Figure 6D**), with the combination of CA125, HE4 and NLR demonstrating the highest specificity. Collectively, these findings suggest that the integration of CA125, HE4 and NLR panels as a screening approach may enhance the effectiveness of early diagnosis in patients with ovarian cancer (**Table 4**).

**Table 4:** Training phase.

	cut-off value	Sensitivity (%)	Specificity (%)	AUC	95%CI	p Value
TNM: I-IV						
CA125	39.570	80.77	92	0.9129	0.8722 - 0.9537	<0.001***
HE4	63.250	76.92	99	0.9408	0.9100 - 0.9717	<0.001***
CEA	1.740	27.88	85	0.5423	0.4628 - 0.6218	0.297
AFP	2.295	50.00	76	0.6450	0.5699 - 0.7201	<0.001***
NLR	2.031	42.31	98	0.7098	0.6376 - 0.7821	<0.001***
CA125+HE4	0.499	83.65	96	0.9494	0.9208 - 0.9780	<0.001***
CA125+HE4+NLR	0.376	81.73	98	0.9544	0.9270 - 0.9819	<0.001***
TNM: I-II						
CA125	39.570	79.17	87	0.8679	0.7998 - 0.9360	<0.001***
HE4	59.240	75.00	87	0.8851	0.8256 - 0.9446	<0.001***
NLR	2.229	22.92	98	0.5598	0.4518 - 0.6678	0.240
CA125+HE4	0.499	68.75	96	0.9006	0.8448 - 0.9565	<0.001***
CA125+HE4+NLR	0.720	81.25	87	0.9083	0.8537 - 0.9630	<0.001***
TNM: III-IV						
CA125	73.280	89.29	95	0.9515	0.9122 - 0.9908	<0.001***
HE4	73.120	91.07	99	0.9886	0.9766 - 1.000	<0.001***
NLR	2.287	67.86	90	0.8384	0.7662 - 0.9106	<0.001***
CA125+HE4	0.494	96.43	96	0.9913	0.9808 - 1.000	<0.001***
CA125+HE4+NLR	0.402	94.64	97	0.9939	0.9869 - 1.000	<0.001***
Low CA125						
CA125	19.950	56.25	63	0.5428	0.4075 - 0.6781	0.583
HE4	55.990	75.00	77	0.7916	0.6683 - 0.9148	<0.001***
NLR	2.686	31.25	91	0.6188	0.4480 - 0.7895	0.128
CA125+HE4	0.820	62.50	80	0.7800	0.6527 - 0.9073	<0.001***
CA125+HE4+NLR	0.777	68.75	80	0.8063	0.6803 - 0.9322	<0.001***
High CA125						
CA125	47.960	96.59	91	0.9802	0.9636 - 0.9968	<0.001***
HE4	65.790	86.36	99	0.968	0.9459 - 0.9900	<0.001***
NLR	2.185	44.32	98	0.7264	0.6503 - 0.8024	<0.001***
CA125+HE4	0.443	92.05	97	0.9802	0.9651 - 0.9953	<0.001***
CA125+HE4+NLR	0.376	92.05	98	0.9814	0.9660 - 0.9967	<0.001***
Low HE4						
CA125	39.570	70.21	87	0.8263	0.7486 - 0.9040	<0.001***
HE4	55.980	82.98	77	0.869	0.8068 - 0.9313	<0.001***
NLR	2.239	27.66	98	0.6068	0.4985 - 0.7151	0.037*
CA125+HE4	0.802	89.36	73	0.8881	0.8295 - 0.9466	<0.001***
CA125+HE4+NLR	0.720	80.85	87	0.8991	0.8423 - 0.9560	<0.001***
High HE4						
CA125	73.280	96.49	86	0.9844	0.9697 - 0.9991	<0.001***
HE4	122.200	100.00	88	1.0000	1.000 - 1.000	<0.001***
NLR	2.254	56.14	91	0.7947	0.7138 - 0.8757	<0.001***
CA125+HE4	0.015	100.00	88	1.0000	1.000 - 1.000	<0.001***
CA125+HE4+NLR	0.017	100.00	100	1.0000	1.000 - 1.000	<0.001***

*p < 0.05; ***p < 0.001.

### Validation of the efficiency of CA125 and HE4 combined with NLR in the validation phases

**Figure 7:**
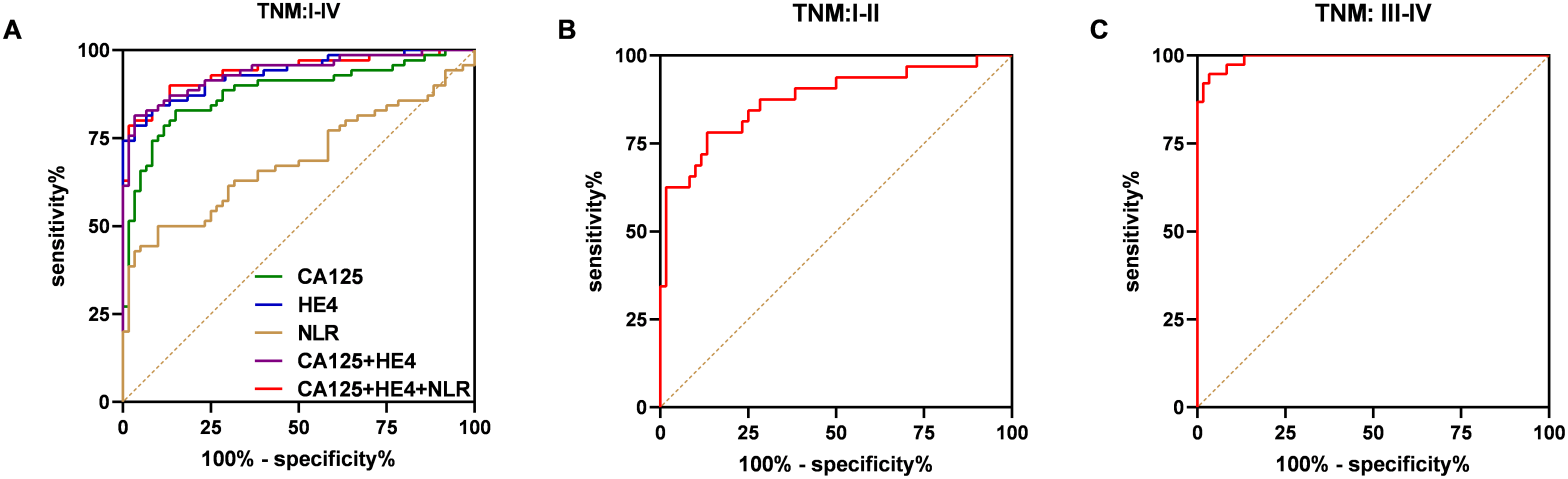
ROC curve analysis of the combination of the CA125, HE4 and NLR based diagnostic model in distinguishing OC cases from controls in the validation dataset. ROC curves of the model for OC cases with overall stages (I–IV) **(A)**, early stages (I–II) **(B)**, advanced stages (III–IV) **(C)**.

**Figure 8:**
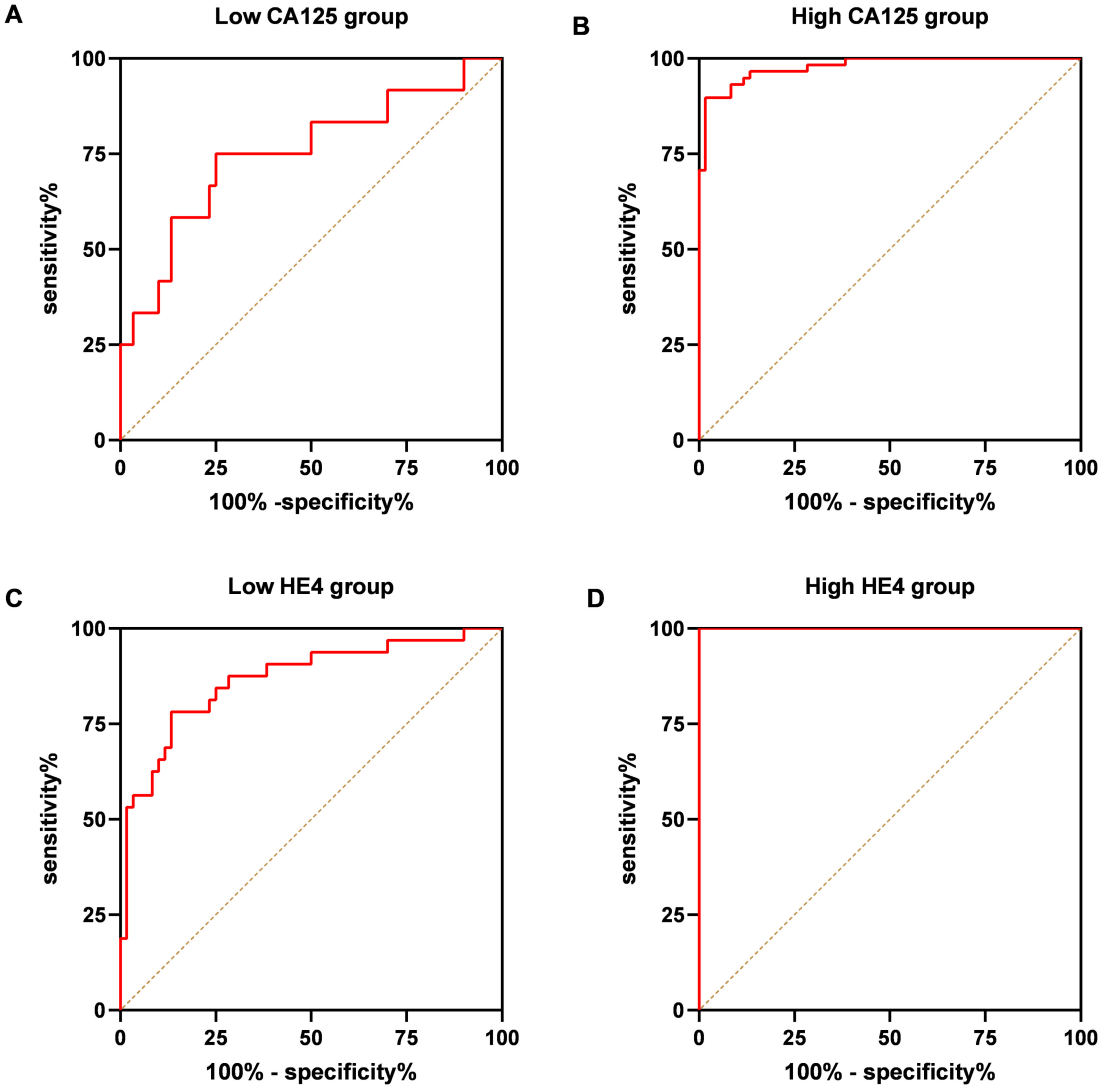
ROC curve analysis of the combination of the CA125, HE4 and NLR based diagnostic model in distinguishing OC cases from controls in the validation dataset. ROC curves of the model for OC cases with low-CA125 level group **(A)**, high-CA125 level group **(B)**, low-HE4 level group **(C)**, high-CA125 level group **(D)**.

The validation of the model was conducted using a separate cohort comprising 70 OC patients and 60 control subjects, which included 30 individuals with benign ovarian tumors and 30 healthy women (**Table 1**). As illustrated in **Figure 7A**, the AUC for the model was 0.9393, demonstrating a sensitivity of 78.57% and a specificity of 98.33%, surpassing the performance of other markers. Specifically, CA125 yielded an AUC of 0.8813 with a sensitivity of 82.86% and a specificity of 85%; HE4 presented an AUC of 0.9351 with a sensitivity of 78.57% and a specificity of 96.67%; the NLR exhibited an AUC of 0.6860, sensitivity of 50%, and specificity of 90%; while the combination of CA125 and HE4 resulted in an AUC of 0.9371, sensitivity of 81.43%, and specificity of 96.67% (**Figure 7A**). Furthermore, within the early-stage (I and II) cohort, the AUC for the panel was recorded at 0.8760, with a sensitivity of 78.13% and a specificity of 86.67% (**Figure 7B**). In contrast, the advanced-stage (III and IV) group demonstrated an AUC of 0.9925, with a sensitivity of 94.74% and a specificity of 96.67% (**Figure 7C**). For instance, as depicted in **Figure 8**, in the low CA125 level (≤35 U/mL) subgroup, the AUC of the panel was 0.7514, with both sensitivity and specificity at 75% (**Figure 8A**). Conversely, in the high CA125 level (>35 U/mL) subgroup, the AUC reached 0.9782, with sensitivity at 89.66% and specificity at 98.33% (**Figure 8B**). In the low HE4 level (≤140 pmol/L) group, the AUC was 0.8672, with sensitivity of 78.13% and specificity of 86.67% (**Figure 8C**). Notably, in the high HE4 level (>140 pmol/L) group, the AUC was perfect at 1, indicating a sensitivity and specificity of 100% (**Figure 8D**). These findings substantiate the hypothesis that the integrated screening protocol using CA125, HE4 and NLR may significantly enhance the effectiveness of early diagnosis in patients with OC (**Table 5**).

**Table 5:** Validation phase.

	cut-off value	Sensitivity(%)	Specificity(%)	AUC	95%CI	p Value
TNM: I-IV						
CA125	40.150	82.86	85.00	0.8813	0.8210 -0.9416	<0.001***
HE4	70.560	78.57	96.67	0.9351	0.8942 - 0.9760	<0.001***
NLR	2.645	50.00	90.00	0.6860	0.5941 - 0.7778	<0.001***
CA125+HE4	0.503	81.43	96.67	0.9371	0.8961 - 0.9782	<0.001***
CA125+HE4+NLR	0.275	78.57	98.33	0.9393	0.8983 - 0.9803	<0.001***
TNM: I-II						
CA125+HE4+NLR	0.720	78.13	86.67	0.8760	0.7946 - 0.9574	<0.001***
TNM: III-IV						
CA125+HE4+NLR	0.402	94.74	96.67	0.9925	0.9822 - 1.000	<0.001***
Low CA125						
CA125+HE4+NLR	0.781	75.00	75.00	0.7514	0.5816 - 0.9212	0.006**
High CA125						
CA125+HE4+NLR	0.276	89.66	98.33	0.9782	0.9574 - 0.9990	<0.001***
Low HE4						
CA125+HE4+NLR	0.720	78.13	86.67	0.8672	0.7843 - 0.9501	<0.001***
High HE4						
CA125+HE4+NLR	0.017	100.00	100.00	1	1. 0000 - 1.0000	<0.001***

**p < 0.01; ***p < 0.001.

### Prognosis evaluation

**Figure 9:**
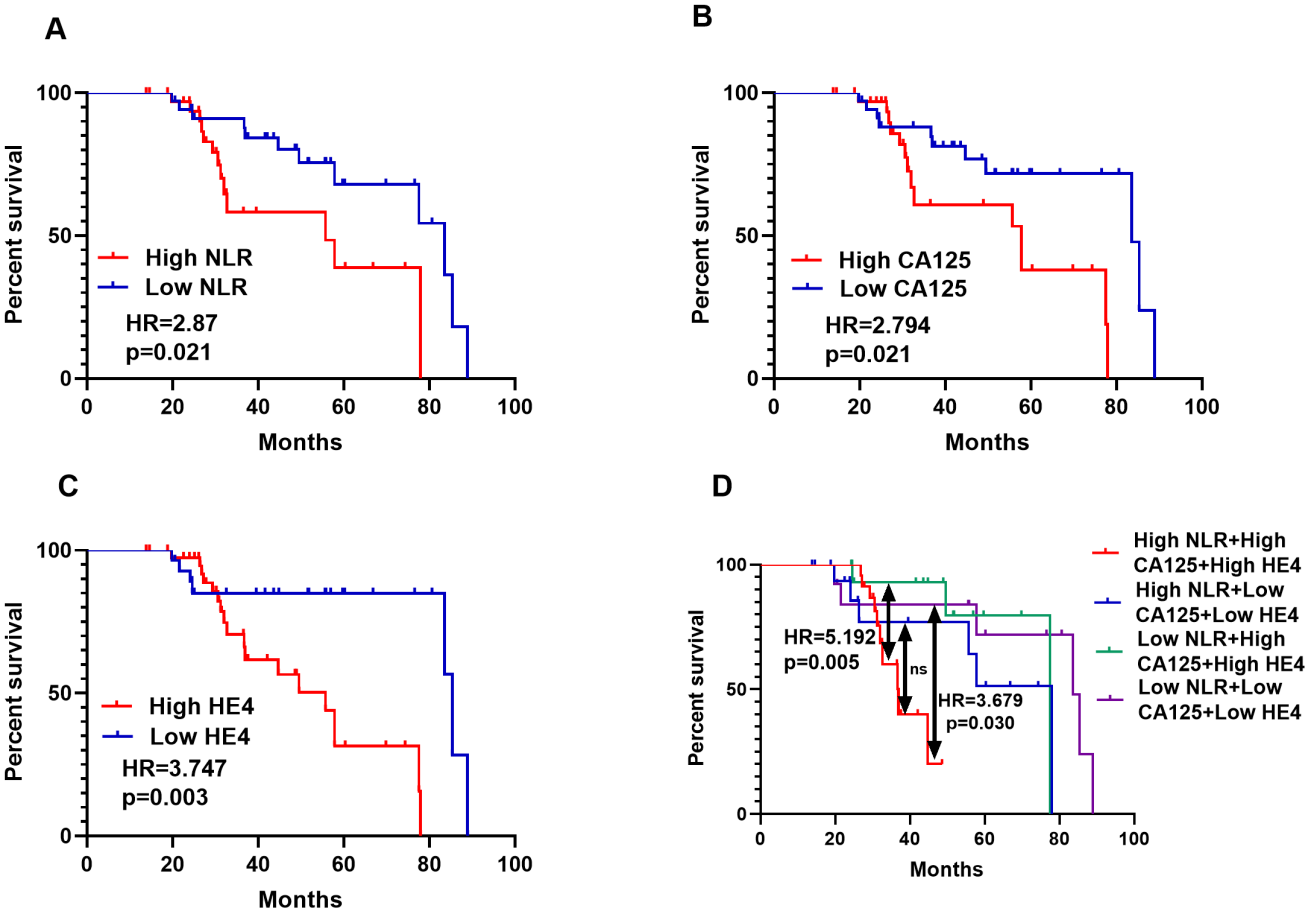
NLR, CA125 and HE4 levels were inversely associated with outcomes of OC patients. Serum NLR and follow-up data of 70 OC patients were analyzed for the correlation between the three biomarkers expression and survival **(A)**, Serum CA125 and follow-up data of 70 OC patients were analyzed for the correlation between the CA125 expression and survival **(B)**, Serum HE4 and follow-up data of 70 OC patients were analyzed for the correlation between HE4 expression and survival **(C)**, Serum NLR, CA125 and HE4 and follow-up data of 70 OC patients were analyzed for the correlation between the three biomarkers expression and survival **(D)**.

The median OS for the full group included in the analysis was 36.6 months. OC patients with lower NLR had a longer overall survival (OS) than OC patients with higher NLR (p = 0.021, **Figure 9A**). The expression levels of CA125 and HE4 were also negatively correlated with the prognosis of ovarian cancer patients (p=0.021, p=0.021, **Figures 9B, C**). Compared with patients with low expression of three biomarkers, patients with high expression of three biomarkers had significantly shorter OS (p=0.030, **Figure 9D**), and patients with high expression of all three biomarkers had significantly shorter OS (p=0.005, **Figure 9D**) compared with patients with low expression of NLR CA125 and high expression of HE4. It shows that the characteristics of the three biomarkers exhibit better prognostic performance than a single biomarker.

As shown in **Table 6**, univariate analysis showed that age (hazard ratio = 1.220,p<0.001), Figo stage (hazard ratio =1.390,p<0.001), N stage (hazard ratio = 2.180, p = 0.001), ascites (hazard ratio = 3.180, p = 0.001), the menopause (hazard ratio = 1.390, p = 0.020), tumor size (hazard ratio = 2.390, p = 0.019), CA125 levels (hazard ratio = 2.179, p = 0.021), HE4 levels (hazard ratio = 3.217, p = 0.012), and NLR (hazard ratio = 6.315, p = 0.003) with poor prognosis related. Interestingly, a multivariate Cox regression analysis showed age (hazard ratio = 1.243,p=0.048),Figo stage (hazard ratio =1.161,p=0.003), CA125 level (hazard ratio =4.711,p=0.0032),HE4 level (hazard ratio =4.550,p=0.038), and NLR(hazard ratio =1.410,p=0.031) were independent prognostic factors for survival in OC patients with OS. In summary, these results suggest that NLR levels in OC patients have significant prognostic value.

**Table 6:** Univariable and multivariate cox regression model analysis.

Characteristics	Univariable model			Multivariable model		
	HR	95%CI	p Value	HR	95%CI	p Value
Age (≥50 vs<50)	1.220	1.09-1.51	<0.001***	1.243	1.06-1.99	0.048*
Figo stage (III-IV vs I-II)	1.390	1.04-1.62	<0.001***	1.161	1.05-2.54	0.003**
N (Yes vs No)	2.180	2.07-2.48	0.001**	2.533	0.15-3.89	0.330
Ascites (Yes vs No)	3.180	2.07-4.48	0.001**	2.468	0.14-3.55	0.214
Menopause (Yes vs No)	1.390	1.18-2.86	0.020*	1.372	0.35-5.38	0.650
Tumor size (≥9cm vs<9cm)	2.390	2.18-3.56	0.019*	0.410	0.15-1.12	0.081
CA125(≥Median vs<Median)	2.179	1.04-2.77	0.021*	4.711	1.14-19.51	0.032*
HE4(≥Median vs<Median)	3.217	3.07-5.72	0.012*	4.550	1.09-19.00	0.038*
NLR(≥Median vs<Median)	6.315	1.84-21.68	0.003**	1.410	1.15-2.12	0.031*

N, lymph node metastases; *p < 0.05; **p < 0.01; ***p < 0.001.

## Discussion

Ovarian cancer is the 5th worldwide leading cause of death of women due to cancer (26). There is poor prognosis in the advanced stages, although there have been great advances in the diagnosis OC in past decades, for example, the application of CA125, HE4, and ROMA, but there have been discrepancies about diagnostic performances, the dire need for improving early detection screening methods exist. NLR was a valuable predictive indicator of various cancer types, there is a growing consensus that effective detection of early-stage cancer will likely rely on biomarker panels that have greater specificity and sensitivity, compared with single biomarkers (27, 28). The results showed that the sensitivity and specificity of the combined detection of NLR and CA125 were significantly higher than that of a single index, suggesting that the combined detection could improve the accuracy of OC diagnosis (29, 30). Tumor is closely related to systemic chronic inflammation, especially in the early stage of tumor metastasis. A small amount of cancer cell metastasis cannot be detected by imaging, but can be reflected by systemic inflammatory indicators, which have been confirmed in cholangiocarcinoma (31). This study found that the level of NLR in the blood of OC patients was significantly higher than that of the benign disease group and the healthy control group, while the expression of NLR in the benign disease group was not statistically significant compared with the healthy control group.

The sensitivity of NLR in the diagnosis of OC was lower than that of CA125 and HE4, but the sensitivity of NLR combined with CA125 and HE4 was higher than that of either single index, and also higher than that of CA125 and HE4, suggesting the value of NLR combined with CA125 and HE4 in the diagnosis of OC and related diseases. After the occurrence of tumor, the immune function of the body is decreased, showing that the number of lymphocytes may decline. In addition, the presence of tumors, including a small number of tumor cell metastases, must lead to systemic inflammatory responses, which also lead to the relative elevation of neutrophils. The relative increase of neutrophils and the relative decrease of lymphocytes inevitably lead to the increase of NLR. It is speculated that if the patient’s NLR is persistently elevated, it means that the tumor is progressing and the postoperative prognosis is poor, which can be a risk prognostic factor for OC. The results also showed that NLR had significant differences in age, FIGO stage, lymph node metastasis and ascites in OC patients. There was no correlation with tumor diameter or menopause. This indicates that the age of patients affects NLR, and the older the age, the higher the NLR value. The expression of CA125 and HE4 is independent of each other, and the distribution of the two in OC patients is significantly different. This study found that the sensitivity of serum CA125 to OC diagnosis (81.0%) was higher than that of HE4(70.7%), but the specificity was lower than that of HE4(88.7% vs 100%). Most studies at home and abroad were consistent with the results of this study, but there were also different results (32). HE4 and ROMA have the highest diagnostic specificity and the best diagnostic efficiency for OC, but are less sensitive (33). The expression of CA125, HE4 and ROMA ratio were closely related to the number of tumors, TNM stage and pelvic mucus in OC patients, but were not related to age and tumor diameter (34). The combined detection of CA125 and HE4, especially the risk prediction model (ROMA ratio) calculated on the basis of the two indicators, combines the advantages of the two, has better diagnostic efficiency, and is complementary to NLR in the diagnosis of OC and prognosis of OC patients.

In this study, we showed that a combination of serum NLR, CA125 and HE4 levels was a novel and effective diagnostic biomarker panel for OC patients. Firstly, we found that the serum concentrations of NLR, CA125 and HE4 in OC patients were significantly higher than those in healthy individuals, and the serum concentrations of NLR, CA125 and HE4 were significantly higher in OC patients with advanced stages, lymph node metastases and ascites. Secondly, NLR had a positive correlation with CA125 and HE4, respectively. The higher expression of NLR, CA125 and HE4 was strongly correlated with advanced clinicopathological factors. Thirdly, the ROC curve analysis showed that a combination of NLR, CA125 and HE4 was a more effective panel than traditional combination of CA125 and HE4 for discriminating OC cases from benign ovarian disease and healthy women. Surprisingly, in the early stage, low CA125 groups and low HE4 groups we achieved the same satisfactory results. Overall, we validated the higher accuracy of a combination of NLR, CA125 and HE4 of OC in patients compared to traditional diagnostic markers.

This study also revealed that the expression levels of NLR, CA125 and HE4 can be used as prognostic biomarkers. Initially, we observed that serum expressions of NLR, CA125, and HE4 were strongly correlated with poor clinicopathological parameters. In addition, their elevated serum levels were associated with poorer survival. Therefore, serum NLR, CA125 and HE4 were used as an independent prognostic factor. Overall, our study suggests that in addition to being potential diagnostic biomarkers, NLR, CA125, and HE4 may also be prognostic predictors of OC.

Although combination of NLR, CA125 and HE4 appear to be promising biomarkers, our current study is limited for several reasons. First, elevated NLR levels may reflect inflammatory conditions, which can affect the effectiveness of disease detection. Secondly, this study was a single-center retrospective analysis. Thirdly, clinical parameters are variable between institutions and/or individual clinicians; hence, the results of this small cohort may reflect biases inherent in the acquisition of such clinical data. Therefore, the validation of combination of NLR, CA125 and HE4 as OC biomarkers will require future prognostic model analysis and large-scale counter screening.

## Conclusions

In conclusion, our study demonstrated that serum NLR combined with CA125 and HE4 improves the diagnostic efficiency in patients with ovarian cancer.

## Data Availability

The raw data supporting the conclusions of this article will be made available by the authors, without undue reservation.
